# Mental Health Interventions for Parent Carers of Children with Autistic Spectrum Disorder: Practice Guidelines from a Critical Interpretive Synthesis (CIS) Systematic Review

**DOI:** 10.3390/ijerph15020341

**Published:** 2018-02-14

**Authors:** Denise Catalano, Linda Holloway, Elias Mpofu

**Affiliations:** 1 Department of Rehabilitation and Health Services, University of North Texas, Denton, TX 76203, USA; Linda.Holloway@unt.edu (L.H.); Elias.Mpofu@unt.edu (E.M.); 2 Department of Educational Psychology, University of Johannesburg, Johannesburg, Auckland Park 2006, South Africa; 3 Department of Rehabilitation Counselling, University of Sydney, Lidcombe, NSW 2141, Australia

**Keywords:** autism, autism spectrum disorder, parent carer, mental health, stress, intervention, systematic review

## Abstract

Parent carers of children with Autism Spectrum Disorder (ASD) often report increased levels of stress, depression, and anxiety. Unmet parent carer mental health needs pose a significant risk to the psychological, physical, and social well-being of the parents of the child affected by ASD and jeopardize the adaptive functioning of the family as well as the potential of the child affected by ASD. This systematic review identifies key qualities of interventions supporting the mental health of parent carers and proposes practitioner-parent carer support guidelines. A search of four databases (Medline, PubMed, PsycINFO, and Social Science Data) was conducted to identify studies that met the following criteria: (1) an intervention was delivered to parent carers of a child with ASD under the age of 18 years; (2) the research design allowed for a comparison on outcomes across groups; and (3) outcome measures of the parent carers’ mental health were used. A total of 23 studies met the inclusion criteria. A critical interpretive synthesis approach was used to produce an integrated conceptualization of the evidence. Findings suggest practitioner guidelines to support the mental health and wellbeing of parent carers should include addressing the parent’s self-perspective taking and skill for real time problem-solving.

## 1. Introduction

Parents who are primary carers of a child affected with Autism Spectrum Disorder (ASD) are often found to experience higher levels of stress and poorer physical health when compared with parents of children of typical development [1,2,3], parents of children diagnosed with other disabilities [4,5], or when compared to the general population [6,7,8,9,10,11]. The parenting stress experienced by parents of a child affected with ASD therefore appears to pose a greater risk to the parents’ psychological and health-related quality of life. ASD is a neurodevelopmental disorder characterized by deficits in social interactions and communication skills, both verbal and non-verbal, restricted interests, and stereotypical behaviors [12]. The manifestation of ASD symptoms may range from mild to severe and vary from individual to individual. It is estimated that 1 in 160 children worldwide (or 62.5 per 10,000) are identified with ASD [13], with Japan reported to have the highest prevalence of approximately 161 children per 10,000 identified with the disorder [14]. In the United States, the number of children identified with ASD has increased from 1 in 150 (or 66.7 per 10,000) children estimated in the year 2000, to 1 in 68 (or 147 per 10,000) in 2016, an increase of 119.4 percent [15]. 

Carers of children diagnosed with ASD, often referred to as “autism”, are generally the parents of the child [16]. These parents are at high risk for “caregiver syndrome” or “caregiver stress”, a condition of exhaustion, anger, rage, or guilt that results from unrelieved caring for a chronically ill dependent [17]. “Caregiver burnout” and “caregiver burden” are additional terms often used to describe a state of physical, emotional, and mental exhaustion that may be accompanied by a change in attitude, from positive and caring to negative and unconcerned, as a consequence of attending to the ongoing demands inherent in caring for a dependent individual [18,19].

Some parents become overwhelmed by the daily hassles and general life stresses they experience parenting a child affected by ASD [20]. A growing body of evidence suggests that parents of a child affected with ASD experience higher rates of depression and anxiety [21,22,23,24,25,26,27], fatigue [28], increased problems with physical health and bodily pain [29], and poorer overall well-being [24] and quality of life [26,30]. Increased parenting stress is attributed to the need to provide constant supervision and assistance to the daily living skills of the child, ongoing sleep disruption, lack of available respite care, and lack of responsiveness by school personnel and related services [31,32,33,34]. Moreover, the behavior of children affected with ASD may be difficult to manage due to behavioral anomalies, including temper tantrums and aggressive, self-abusive, destructive, obsessive, ritualistic, impulsive, and self-stimulating behaviors that can pose potential physical harm to the parent carer as well as siblings or family members and friends [8,26,35]. There is a high likelihood that a child affected by ASD may also be affected by a comorbid condition, such as specific phobia (SP), obsessive-compulsive disorder (OCD), or attention-deficit hyperactivity (ADHD), adding to difficulties for parent carers tasked with trying to manage the child’s behavior [36]. Externalizing behaviors (e.g., hyperactivity and conduct problems) have consistently been found to explain a significant portion of parental distress and poor physical health [37].

Addressing the unmet mental health needs of parent carers with a child affected by ASD is necessary in order to improve the overall health and quality of life of the parents, as well as those of other family members [38]. Moreover, improving the parents’ mental health and wellbeing enhances the potential of the child to also achieve a better quality of life. Improvements to the child’s overall behavior and functioning is facilitated when behavioral treatment interventions being delivered to the child are supplemented by continued implementation by the parent carers in the home environment [39]. The effectiveness of the child’s behavioral treatment interventions have been reported to be diminished when parents’ mental health needs are unmet [40].

The health-related quality of life of parent carers of a child affected by ASD may be enhanced by directly reducing parental stress through the engagement of parent carers in effective mental health support strategies. Practice guidelines for supporting the mental health wellbeing of parent carers of children with ASD are important for translating public health care services received by their children into real, sustainable health benefits for them, their families, and their community. Currently guidelines to support the mental health of parents who have a child affected by ASD do not exist, yet these guidelines would be important to identify in order to promote the delivery of cost-effective services to parents who need assistance. Identifying evidence-supported parent carer mental health interventions can provide practitioners, and their parent carer partners, with working guidelines for delivering mental health support services that, upon further development, could be adopted by state and federal agencies with the responsibility for providing services to children affected by ASD and their families and parents.

### Objectives of Study

We specifically aimed to identify and critically cross-walk findings from the extant studies with the intention to propose practice guidelines for practitioners and parent carers for improving the mental health and psychological wellbeing of those parents who have a child affected by ASD and are challenged with maintaining their own wellbeing. Our goal was to apply a critical integrative synthesis analysis to collate the emerging evidence from a diverse body of literature examining mental health supports for parent carers of a child affected by ASD. In sum, we synthesized the nature and results of mental health intervention studies delivered to parent carers of a child affected by ASD reported in studies that examined the effectiveness of mental health interventions as well as qualitative reports in order to provide health and allied-health professionals with practice guidelines that could be implemented into a standard practice of care to address the mental health needs of parent carers of a child affected by ASD.

## 2. Method

### 2.1. Critical Interpretative Synthesis

Critical Interpretive Synthesis (CIS) is a systematic review that allows for triangulation of the evidence gathered from effectiveness (i.e., quantitative) and qualitative studies into new conceptual constructs, grounded in evidence and resulting from a critical interpretation of that evidence [41,42]. The synthesis results in the development of synthetic constructs, transforming the underlying evidence into new conceptual constructs and allow for various aspects of an identified phenomenon described in a diverse body of evidence to be expressed in a more comprehensive and useful way. These synthetic constructs, formed from the critical integration of findings across all studies included in the review, can then be organized into a coherent explanatory theoretical framework [42]. A CIS review of the broad and complex literature that intersects parenting, mental health, and ASD can provide more useful and pragmatic practice guidelines for practitioners and parent carers than what can be produced from conventional reviews alone (e.g., meta-analyses).

### 2.2. Search Criteria and Literature Search

The focus of the synthesis reported in this review was on interventions provided to parents of a child with ASD that resulted in the parent carers improved mental health. Studies included in this review met the following inclusion criteria: (1) an intervention was delivered directly to one or both parent carers of a child with ASD under the age of 18 years; (2) the study involved a research design allowing for a comparison across groups on the outcome of an intervention; (3) an outcome measure of parental mental health was used; and (4) the study was published in English in a peer-reviewed journal. Parent carers were defined as maternal mothers or paternal fathers who resided in the same home as their child with ASD and who had responsibility for the child’s care. “Mental health” was defined in accordance with the World Health Organization definition of mental health—“a state of well-being in which every individual realizes his or her own potential, can cope with the normal stresses of life, can work productively and fruitfully, and is able to make a contribution to her or his community” [43] (para. 1). Outcome measures included any instrument that assessed some aspect of mental health, including measures of stress, anxiety, depression, quality of life (including health-related quality of life), and subjective well-being. There were no publication date restrictions applied, and other systematic reviews or review-based articles were not included.

### 2.3. Search Procedure

A systematic search of the databases Medline, PubMed, PsycINFO, and Social Science Data was conducted using key search terms identified from relevant papers found in a preliminary search, and from a search of relevant Medical Subject Headings (MeSH) terms which were then incorporated into an expanded search strategy. The MeSH and text words for Autism (Autism, Autism Spectrum Disorder, ASD, developmental disorder, Asperger’s Syndrome, Asperger’s) were combined with MeSH and text terms for parent carers (mother, father, caregiver, carer, maternal, paternal), MeSH and text words for intervention (intervention, support, effects, improve, train, treatment, program), and MeSH and text words for mental health (mental health, mental wellbeing, psychological wellbeing, wellbeing, stress, depression, anxiety). Boolean “OR” was used to connect search terms within each set of text words, while “AND” was used to connect the four sets of text words. Terms that had plural forms (e.g., parents) were truncated (i.e., “*”) and hyphenated words (e.g., well-being) were entered in both their hyphenated and non-hyphenated form. Since both quantitative and qualitative studies were desired, no qualifier was entered for research design.

The search was conducted from 15 May through 15 July 2017 by the primary author. Citations identified by the search were exported to the first author’s RefWorks account for de-duplication. The first and second co-authors assessed remaining studies for relevance by screening titles and abstracts, discarding those that did not meet the inclusion criteria. Full-text versions of the retained studies were obtained and examined independently by the first and second co-authors for study eligibility. Reference lists of the retained articles were hand searched for potential additional articles. Additional articles found were reviewed in their full-text version by the first two-co-authors to ensure they met the eligibility standards. Inter-observer agreement of the retained studies was 100%.

### 2.4. Quality Appraisal

A quality appraisal of each qualitative study was conducted prior to the study being included in the final review and synthesis. The quality of a qualitative study is largely determined by the quality of the published report since there is no consensus on a set of specific standards [44]. The individual studies included in the present integrative synthesis were assessed using a published checklist [45] that identifies nine specific elements of the published report, each evaluated using a four-point Likert-type scale (4 = good, 3 = fair, 2 = poor, 1 = very poor). The nine elements included: (1) the abstract and title, (2) introduction and aims, (3) method and data, (4) sampling procedure, (5) data analysis, (6) ethics and bias, (7) findings and results, (8) transferability/generalizability, and (9) implications and usefulness. Scores for each article are summed with higher scores reflecting higher methodological quality (scores range from 9 (very poor) to 36 (very good). The first author assessed the quality of each study retained in the search and found the majority of the studies were of good methodological quality, with scores ranging from 24 to 35. The 2nd co-author randomly selected 30% of the studies and conducted an independent assessment. No significant disagreements occurred (scores between co-authors differentiated by no more than one or two points on average) and discrepancies were discussed until consensus was achieved. Both co-authors agreed that one particular study should be excluded based on having a score substantially lower than the other studies (score = 14) due to several noted and significant methodological limitations.

## 3. Results

### 3.1. Search Results

**Figure 1:**
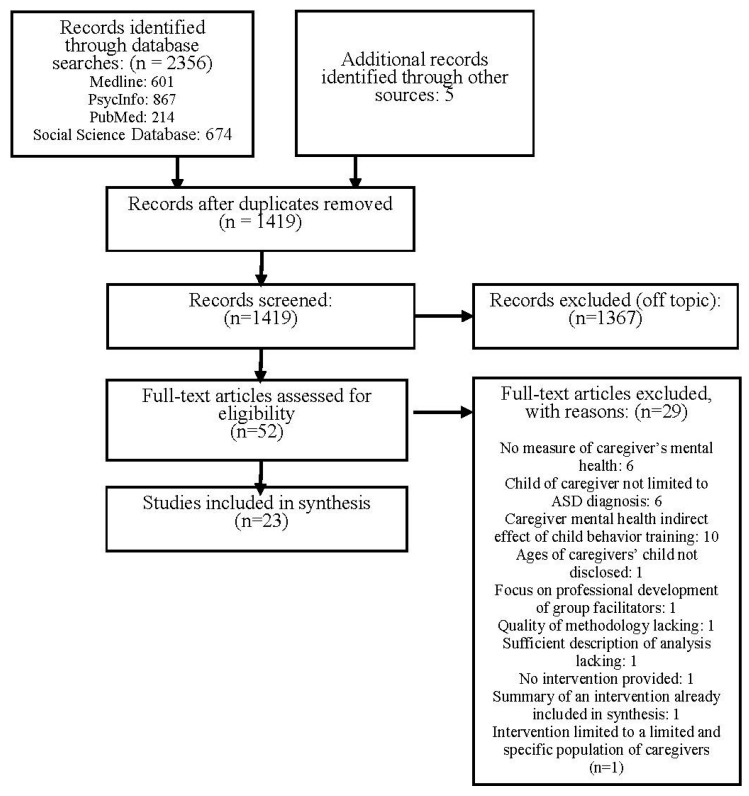
Flowchart of study selection process.

Figure 1 provides the number of total identified citations and results of the eligibility screening process. The search yielded 2361 citations, with 942 citations determined to be duplicates. Of the remaining 1419 records, 1367 records were deemed ineligible following a review of the title and abstract information. Full-text articles were obtained of the remaining 52 studies and reviewed independently by both the first and second co-authors. A total of 29 studies were excluded by mutual consensus of the co-authors (see explanations provided in Figure 1) resulting in a total of 23 studies included in the synthesis. 

### 3.2. Study Characteristics

Table 1 describes specific characteristics of the 23 studies included in this synthesis (authors and reference, country of origin, aim, design, data collection, analysis method, psychological construct(s) measured, measurement instruments, participant information, and the study’s findings). The included studies reported outcomes for a total of 918 participants, with at least 70% being the mother of the child (two studies did not provide gender characteristics). Six studies were based on an experimental design with random assignment of participants to a control or treatment group, with the remaining studies using either a quasi-experimental pre-post design (*n* = 10), or mixed-methods design (*n* = 7). Studies included in the review were conducted in a total of 13 countries: Australia (*n* = 3), Canada (*n* = 1), China (*n* = 2), Greece (*n* = 1), India (*n* = 1), Iran (*n* = 3), Japan (*n* = 1), Jordan (*n* = 1), South Korean (*n* = 1), Spain (*n* = 1), Turkey (*n* = 1), United Kingdom (*n* = 1), and the United States (*n* = 6). 

### 3.3. Data Extraction

In order to ensure that none of the key aspects of the studies were overlooked, a grid was developed (see Table 2) that allowed for the recording of key concepts from each study based on the nature of the intervention, results of the study, perspectives reported by the participants, and the conclusions and observations of the investigators. Columns were constructed with the top row identifying the study’s citation number, while the rows of the grid listed the key concepts drawn from each study. The concepts were then integrated across studies through a series of discussions among the co-authors regarding the similarities and differences of the individual concepts within the context of the various studies, producing a reduced number of transformed synthetic concepts which were then grouped together into a structure of higher order themes. These overarching, higher order themes identified a synthesized interpretation representative of the whole body of evidence.

### 3.4. Synthesis

Three major themes were identified as being central to improving the mental health of parents who have a child affected by ASD—access to social support with similar parent carers, receiving professional stress management and problem-solving training; and the provision of accurate information regarding ASD. Table 2 lists the major themes and their subthemes resulting from the integrated synthesis of evidence reported in the individual studies. The top row indicates the citation for the study.

#### 3.4.1. Social Support

The role of social support was found to be an important factor in twelve studies that either specifically examined the role of social support in improving the psychological wellbeing of parents or included a group treatment condition in which parents were able to engage in discussions with other parent carers. The theme of social support encompassed the roles of informal networks, reduced isolation, and validation by similar parent carers.

##### Informal Networks

Seven studies [46,56,65,101,103,113,116] involved an intervention examining the role of social support and found parent carers involved in a parenting social group also reported decreased anxiety [65] and social stress [103,113], increased group cohesion [46], and improved health and family functioning [113] and quality of life [101]. The benefits of a parenting support group on measures of parental well-being was found to be maintained up to twelve months post intervention [115]. An asynchronous on-line support group appeared to not to be effective in reducing parenting stress, anxiety, or depression [56], suggesting the beneficial effect of social support may involve direct, real-time communication methods (e.g., face-to-face, telephone calls). 

##### Reduced Isolation

Five studies found social support related to improvements in parents’ psychological wellbeing was attributed to the increased awareness by parents that they were not alone in the challenges they faced parenting a child affected by ASD. Parents who reported feeling less socially isolated because of having access to other parents who were also engaged in the intervention reported a decrease in anxiety and stress [46,61,65,113,116].

##### Validation by Peers

Feedback from parents in eight studies [46,61,65,77,92,113,116,117] suggests that the validation they received from the other parents as an important factor to improvements of their wellbeing. These studies found social support was associated with increased group cohesion [46], confidence [65], and post-traumatic growth [117], as well as less hopelessness [61], stress [77,78,113], anxiety [61], depression [116]. The normalizing effect due to receiving support and empathy from other group members was reported by parents as a factor in facilitating their ability to cope and adjust their perspective to the parenting challenges they faced. Parents were able to recognize that their experience was not all negative but rather a different “normal”. 

#### 3.4.2. Professional Training in Skill Development

Fourteen studies included professionally-led workshops based on cognitive-behavioral approaches that provided parents with various stress management strategies (nine studies), and problem-solving skill training (six studies).

##### Stress Management Strategies

Nine studies [46,49,61,70,75,92,96,105,108] involved interventions that provided training in various stress management strategies. Biofeedback was found to increased group cohesion and reportedly provided parents with a helpful strategy for dealing with their day to day stress [46], while mindfulness was associated with decreased mood disturbances [111], and increased psychological [92,105] and general health [70], as well as overall quality of life [105]. Progressive muscle relaxation was associated with decreased stress [75], and expressive writing, particularly writing related to describing the benefits of being a parent carer, was associated with decreased anxiety [96]. Interventions incorporating Acceptance and Commitment therapy, found the approach associated with decreased depression and distress, and increased psychological flexibility [49,89].

##### Problem-Solving Skills

Five studies [61,65,66,98,117] involved parents receiving training in problem-solving or coping strategies. These interventions were reported to be particularly useful to parents when the training was structured and focused on providing practical knowledge and skills for dealing with their child’s behavioral problems and daily care. Skill training in problem-solving strategies appeared to be associated with parents’ increased use of social support [61,66], increased confidence in addressing problems [65], promoted post-traumatic growth [117], and decreased parent carers’ levels of anxiety [65], stress [66], and depression [98].

#### 3.4.3. Gaining Knowledge Regarding ASD

Eight studies [61,65,72,77,78,103,113,116] examined the influence of providing parent carers with information about ASD and the types of resources and services that were available to them as a strategy for reducing parental stress and anxiety.

##### Understanding Autism Spectrum Disorder

Five studies [65,77,78,113,116] involved an intervention in which parents were provided with information regarding ASD and its related cognitive, emotional, and behavioral features. As parents’ knowledge of ASD increased, stress, anxiety and distress was reduced [65,77,78,116], confidence and ratings of health increased [65,113], and the use of problem-solving skills increased [113].

##### Resources and Services

Four studies [65,72,78,103] involved interventions providing parents information about available resources and services from healthcare professionals as a strategy to address parental stress and anxiety. Parents who were provided with information regarding advocacy services, such as available educational, developmental, and behavioral treatment programs, and had assistance in identifying ways to fill service gaps reported a decrease in anxiety [65,78], stress [103], and were less distressed upon receiving their child’s diagnosis [72].

## 4. Discussion

This systematic review applied a critical interpretive synthesis analysis that included both quantitative and qualitative studies in order to triangulate emerging evidence, and to formulate needed practice guidelines for health and allied-health professionals who provide mental health support to parent carers of a child affected by ASD. Three broad themes emerged from this synthesis of primary studies—the importance of social support by other parent carers, the effectiveness of training parents in stress management strategies and developing problem-solving skills, and the importance of providing parent carers with relevant and accurate information about ASD and available resources and support services. These themes can be effectively incorporated into strategies practitioners and their parent carer partners can utilize for improving not only the psychological well-being of parent carers, but also to influence the wellbeing of the child affected by ASD and other family members.

Perhaps one the most effective factors identified in the synthesis found to influence the well-being of parent carers was being engaged with other parent carers, such as through a parenting social support group. Networking with other parent carers allowed for parents to realize they were not alone in the challenges they faced [56] and provided parents with an important validation of their own value and experiences [56,61,65]. They could share stories with others who may have had similar experiences and discuss life’s difficulties [46,61,65,66], exchange ideas and information about resources [46,61], and learn from other parents how they respond and cope with their child’s behaviors, as well as the criticism often received from others regarding their child’s behavior [46,61]. Through the support of other similar parent carers, parents are able to normalize their experience and become more aware of their child’s needs rather than focusing on a comparison of their child to other children [65,77,92,113]. Receiving validation from others helped increase parent carers’ confidence to cope with daily challenges and be more accepting of their child’s behaviors [46,92]. Parents could also notice the positive changes in each other and provide continued support in the growth and well-being of the other parent carers [56].

Participating in professionally-led workshops in which parent carers were introduced to the practice of various stress management strategies (e.g., biofeedback, mindfulness), acceptance, and learned problem-solving skill strategies, had a positive effect on parents’ mental health and psychological well-being [46,49,61,70,75,89,92,96,105,108]. Helping parents experience the practice and value of “being present”, rather than ruminating over unresolved events in the past, or activating thoughts about anticipated future events, enhances the parents’ ability to stay focused on the present situation in a non-judgmental way and promotes an awareness of choices the parent carer may have in response to a situation. Coupled with training in problem-solving strategies, providing training in stress management strategies has the potential to improve parents carers’ wellbeing by increasing their confidence in effectively managing daily challenges, and their ability to be more accepting of others, including their child as well as themselves. 

Workshops providing training in stress management strategies and problem-solving skills also functioned as social support groups, providing participants with opportunities to develop and practice new problem-solving strategies to the challenges they routinely faced while also learning from other group members about their experiences and strategies. Overall, stress management strategies helped parent carers learn a beneficial strategy for managing stress they were able to practice in their own home and utilize whenever they found themselves facing a difficult situation. Additionally, workshops provided parent carers with an opportunity for respite from their daily caregiving responsibilities.

Parent carers reported that receiving factual and accurate information regarding ASD helped decrease their feelings of stress and anxiety, often expressing relief after receiving information that helped explain their child’s behaviors. Providing parents with a better understanding of how their child processed sensory information, and the relationship of that experience to the child’s engagement in certain behaviors, was reported as making a difference in how parents related and reacted to their child, promoting better acceptance and understanding of the child behavior. When provided with an explanation that related their child’s diagnosis to the child’s behavior, parents’ confidence in seeking appropriate services increased. With accurate and relevant knowledge about the nature of ASD, parents described being able to develop more effective strategies and tools that helped their child better cope with the sensory stimulation they experienced in their environment. Learning about the sensory aspects of autism and strategies that would help to support their child was reported as being highly valued by parent carers.

When developing a treatment plan to address the mental health needs of a parent carers with a child affected by ASD, including any, or all, of the above strategies, should be considered. Improved mental health and psychological well-being was reported by parent carers as a result of each of these strategies, often with strategies, such as receiving social support from similar parent carers, being imbedded into other strategies, such as group workshops on stress management and problem-solving. These strategies can form the framework for the development of strategies that could be delivered to parent carers of a child affected with ASD and perhaps expanded to address parent carers of children with other forms of neurodevelopmental disorders and chronic illness.

### 4.1. Strengths and Limitations of the Review

This review used a rigorous review method for a critical integrative synthesis involving a comprehensive search of published studies examining the effectiveness of an intervention designed to improve the mental health and psychological wellbeing of parents of a child affected with ASD and that met pre-determined inclusion criteria. Key factors were identified from a diverse body of literature that included both quantitative effectiveness studies as well as qualitative studies that provided the participants’ perspectives regarding the benefits and limitations of the interventions, as well as their own beliefs, values, and feelings about their experience with the intervention. Although each study occurred within a different context, we were able to integrate and synthesize findings across primary studies in order to identify higher level constructs that could provide a more comprehensive understanding of the factors involved in effective interventions for addressing the psychological well-being of parents with a child affected by ASD.

It is acknowledged that the results of this study should be considered a beginning step in building a body of knowledge that would prove useful to health and professional mental health practitioners who provide support services to parent carers and their child affected by ASD. In spite of conducting a comprehensive search of the evidence, a synthesis of all forms of evidence is the desired standard, such as unpublished studies, dissertations, single-case studies, and conference presentations, to name a few. Such an undertaking, however, was not feasible given the time and number of personnel involved, thus it was necessary to limit the sampling of evidence to those that met our pre-determined criteria with the hopes that others will continue to expand upon the evidence we have provided.

It is also possible that others might have reviewed the studies included in the present synthesis and identified alternative overarching constructs. The purpose of a critical integrative synthesis is to generate theory [42], and we hope our findings will prove useful in the development of a theory that provides strong explanatory power regarding those factors that must be present in an effective, and efficient, strategy for enhancing the psychological well-being of parents who are experiencing significant distress due to the challenges of parenting a child affected by ASD. For example, there is a paucity of research examining the mental health experience of those parents who live in rural areas and have less access to resources, or are of a lower socioeconomic status, or belong to a minority ethnic population. Additional qualitative research is needed to provide the valuable perspective and insights of parent carers, which should lead to developing randomized controlled studies in order to compare intervention strategies and determine what works for whom and under what circumstances.

### 4.2. Implications of the Review and Practice Guidelines

This synthesis identifies several strategies that could be implemented by practitioners immediately in order to improve the mental health of parent carers of a child affected by ASD. First, incorporating an opportunity for parents to interact with other similar parents appears to be a very beneficial strategy for enhancing parents’ psychological wellbeing. Facilities providing behavioral services to children with ASD should consider integrating support services for parents, such as forming parental support groups, in addition to the behavioral services being delivered to the child affected by ASD. Having a practitioner who is knowledgeable about ASD, and familiar with the issues and challenges parent carers face caring for a child affected by ASD, lead or participate in the support group would be beneficial so that parents could ask questions and obtain relevant and accurate information about ASD. Many parents may lack available child care and having a parent support group available at the facility where their child is receiving behavioral services may be the parent’s only opportunity for beneficial social support and access to information. Parents should be encouraged to form relationships with the other parents so that the provision of peer support could potentially continue as the child ages and parents face new challenges. Practitioners could develop and lead parent support groups that include activities and training in various skills to enhance stress management, coping, and problem-solving practices. Incorporating parent support services formerly into the services being provided to the child at the treatment facility could be an important factor for facilitating the success of the child’s treatment. Secondly, practitioners could serve as advocates for parents and help them find appropriate services and resources to ensure there are no gaps in services for the child. Health and mental health professional practitioners should discuss with parent carers the different aspects of their quality of life and ensure the parent is referred for appropriate services. Thirdly, health and mental health practitioners should provide parents with written information regarding the nature of ASD that could be shared with other family members, as well as guidelines for problem-solving, strategies for stress management, and available resources and services that might be beneficial for either the child or the parent. Treatment facilities that deliver behavioral interventions for children with ASD might consider integrating these described strategies into a package of mental health support services that parent carers could receive, ensuring that the wellbeing of both the child and parent carers are being effectively addressed.

## 5. Conclusions

Parents providing ongoing care to a child affected by ASD are found to be at higher risk for adverse mental health outcomes such as chronic stress, depression, and anxiety. Practice guidelines for the use by practitioners and parent carers of children with ASD should address the need for engaging with other similar parent carers, and enhance their generic problem-solving abilities, self-perspective taking, and sense of meaning as carers. Practitioners who address the state of the parent’s mental health and psychological well-being will enhance the health-related quality of life of the parent carers, their families and their child with ASD. The implementation of the guidelines proposed in this integrative synthesis can potentially lead to greater/better cooperation with child care services, as well as an improved quality of life for parent carers and their child.

## Figures and Tables

**Table 1:** Characteristics of studies included in the synthesis.

Reference, Year, Country	Aim	Design/Data Collection/Analysis Psychological Construct Measured: Instrument	Participants	Findings
Bitsika and Sharpley [46], 2000, Australia	To explore effect of parent support program providing specific stress management strategies	Mixed design, within-subject/questionnaires, written feedback/MANOVA content analysis Anxiety: Self-Rating Anxiety Scale (SAS; Zung [47]) Depression: Self-rating Depression Scale (SDS; Zung [48])	*n* = 11 mothers	No significant change in stress, anxiety, or depression but measure of group cohesion increased; parent’s written feedback supported benefits gained from learning specific stress management technique (biofeedback)
Blackledge and Hayes [49], 2006, United States	To determine effectiveness of Acceptance and Commitment Therapy (ACT) on parent carers’ coping and mental health	Within-subject, repeated measures/questionnaires/Non-parametric Wilcoxon signed-ranked test Acceptance: Acceptance and Action Questionnaire (AAQ; Hayes, Strosahl, et al. [50]) Cognitions and automatic thoughts: Automatic Thoughts Questionnaire (ATQ; Hollon and Kendall [51]) Depression: Beck Depression Inventory II (BDI-II; Beck, Steer, and Brown [52]) General health: General Health Questionnaire-12 item (GHQ-12; Goldberg [53]) Locus of control: Parental Locus of Control Scale (PLOS; Campis, Lyman, Prentice-Dunn [54]) Psychological distress: Global Severity Index (GSI) from the Brief Symptom Inventory (BSI; Derogatis and Melisaratos [55])	*n* = 20 (15 mothers; 5 fathers)	Improved psychological outcomes retained at three month follow-up, general distress and depression decreased significantly, was most pronounced among participants in clinical range of depression
Clifford and Minnes [56], 2013, Canada	To investigate changes in parent well-being following involvement in on-line support group	uasi-experimental between-group/questionnaires/MANOVA Anxiety: State-Trait Anxiety Inventory (STAI; Spielberger [57]) Depression: State-Trait Depression Scales (STADS; Spielberger et al. [58]) Family functioning: Kansas Inventory of Parental Perceptions (KIPP; Behr et al. [59]) Perceived stress: Family Stress and Coping Interview (FSCI; Nachshen et al. [60])	*n* = 45 (43 mothers, 2 fathers) treatment group: *n* = 20, control group: *n* = 25	No significant differences on parenting stress, anxiety, or depression
Erguner-Tekinalp and Akkok [61], 2004, Turkey	To explore effectiveness of coping skills training on stress, coping skills, and hopelessness among mothers	Mixed method, between-group/questionnaires, interviews/Mann Whitney U Test, content analysis Coping: Coping Strategy Indicator (CSI; Amirkhan [62]) Hopelessness: Beck Hopelessness Scale (BHS; Beck, Lester, and Trexler [63]) Parenting stress: Questionnaire on Resources and Stress (QRS; Holroyd [64])	*n* = 20 mothers; Treatment group: *n* = 10, control group: *n* = 10	No significant difference on stress level, but mothers in treatment group reported an increase in use of social support as a coping strategy, and felt a lower sense of hopelessness
Farmer and Reupert [65], 2013, Australia	To decrease parent carers’ anxiety and increase confidence by improving knowledge of ASD	Mixed method, within-subjects/questionnaires/paired *t*-test, inductive thematic analysis, Self-efficacy and parenting anxiety: Understanding Autism and Understanding my Child with Autism (Farmer and Reupert [65])	*n* = 98 (63 mothers, 23 fathers); 86 participants were parents of child, 12 were other famly members	Parental knowledge of ASD, and confidence significantly increased, along with a significant decrease in anxiety
Feinberg et al. [66], 2013, United States	To explore if maternal stress and depressive symptoms would be reduced as the result of strengthening problem-solving skills	Experimental design/questionnaires, interviews/comparison of mean scores, Chronbach alpha Coping: Brief Coping Orientation to Problems (BCOP; Carver [67]) Depression: Quick Inventory of Depressive Symptomatology (QIDS; Rush et al. [68]) Parenting stress: Parenting Stress Index-Short Form (PSI-SF; Abidin [69])	*n* = 110 mothers; Treatment group: *n* = 59, Control group: *n* = 61	At three-month follow-up, mothers in the problem-solving education group reported higher use of social coping (both instrumental and emotional), and were significantly less likely than mothers in the control group to report clinically significant stress and depressive symptoms. No change in using problem-focused or avoidance coping skills
Ferraioli and Harris [70], 2013, United States	To evaluate benefits of mindfulness-based training approach compared to skills-based parentental training program on parental stress	Experimental design, between-group, within-subjects/questionnaires/independent *t*-tests, paired samples *t*-test General health: General Health Questionnaire-28 item (GHQ-28; Goldberg and Williams [53]) Mindfulness: Mindful Attention Awareness Scale (MAAS; Brown and Ryan [71]) Parenting stress: Parenting Stress Index-Short Form (PSI-SF; Abidin [69])	*n* = 15 (10 mothers, 5 fathers) Mindfulness-based group: *n* = 6, Skills-based group: *n* = 9	Parents in mindfulness-based group had significant improvement over skills-based group on measures of parental stress and general health at conclusion of training, and improved general health at 3-month follow-up
Giarelli et al. [72], 2005, United States	To refine a parent-focused nursing intervention and examine effects of post-diagnosis nursing intervention on parental psychological distress and use of services	Mixed methods, pretest-posttest experimental design, observations and survey/questionnaires, interview/non-parametric Wilcoxon test, content analysis Perceived stress: Perceived Stress Scale (PSS; Cohen and Williamson [73]) Psychological distress: Impact of Events Scale (IES; Zilberg et al. [74])	*n* = 31 (16 mothers, 15 fathers), treatment group: *n* = 18, control: *n* = 13	No significant differences on measures of parental distress but parents in intervention group reported being less upset due to unexpected event of child’s diagnosis, the intervention group increased their use of services when compared to treatment-as-usual group (control group).
Gika et al. [75], 2012, Greece	To examine impact of progressive muscle/breathing relaxation intervention on reduction of parental and perceived stress	Within-subjects, repeated measures/questionnaires/non-parametric Wilcoxon signed-rank test for dependent samples Parenting stress: Parenting Stress Index-Short Form (PSI-SF; Abidin [69]) Perceived stress: Perceived Stress Scale-14 items (PSS-14; Cohen, Kamarck and Mermelstein [76])	*n* = 11mothers	Both parental stress and perceived stress was significantly reduced following intervention
Izadi-Mazidi et al. [77], 2015, Iran	To examine the effectiveness of a cognitive-behavior group therapy intervention on parenting stress	Within-subjects, repeated measures/questionnaires/Independent samples *t*-test Parenting stress: Parenting Stress Index-Short Form (PSI-SF; Abidin [69])	*n* = 16 mothers	Parenting stress and distress was significantly reduced after intervention, which included education about ASD, discussions on cognitions and cognitive errors, and the practice of relaxation
Jamison et al. [78], 2017, United States	To evaluate a Family Peer Advocate (FPA) model on improving parent carers’ utilization of services, knowlege of ASD, and sense of empowerment, and reducing parenting stress	Experimental design/questionnaires/Repeated measures ANOVA Caregiver stress or burden: Caregiver-Strain Questionnaire (CSQ; Brannan et al. [79]) Family functioning: Family Empowerment Scale (FES; Karen et al. [80]) Parenting stress: Parenting Stress Index-Short Form (PSI-SF; Abidin [69]) Social support: Social Support Survey (SSS; Sherbourne and Steward [81])	*n* = 39 racial/ethnic minority parents; Treatment group (i.e., FPA assigned to family): *n* = 19 Control group: *n* = 20	Parenting stress in the treatment group was significantly decreased as compared to control group. No change was noted in caregiver’s sense of empowerment, or use of services, although a lack of available services in the community was noted. Parent knowledge of ASD in both groups increased
Ji et al. [82], 2014, China	To determine effectiveness of multi-disciplinary parent education program on improving health-related quality of life (HRQOL) of parent carers	Quasi-experimental between-groups/questionnaires/independent *t*-test and paired samples *t*-test Caregiver stress or burden: Caregiver Burden Index (CBI; Novak and Guest [83]) Coping: Simplified Coping Style Questionnaire (SCSQ; Wang, Wang, and Ma [84]) Family functioning: McMaster Family Assessment Device (FAD; Epstein, Baldwin and Bishop [85]) General health: Short Form Health Survey-36 items (SF-36, Rand Health [86]) Self-efficacy: General Self-Efficacy Scale (GSE; Jerusalem and Schwarzer [87]) Social support: Multidimensional Scale of Perceived Social Support (MSPSS; Zimet, Powell, Farley, Werkman, and Berkoff [88])	*n* = 42 (38 mothers, 4 fathers) Treatment group: *n* = 22, Control group: *n* = 20	Significant improvement in mental HRQOL, family functioning, self-efficacy, and positive coping style were reflected among participants in the intervention group
Joekar et al. [89], 016, Iran	To determine effectiveness of Acceptance and Commitment Therapy (ACT) on Iranian parent carers’ coping and mental health	Quasi-experimental between-group design/questionnaires/MANCOVA Acceptance: Acceptance and Action Questionnaire II (AAQ-II; Bond et al. [90]) General health: Short Form Health Survey-12 items (SF-12, Rand Health [91])	*n* = 24 mothers; treatment group: *n* = 12, control group: *n* = 12	ACT was found to be effective in decreasing symptoms of depression and increasing psychological flexibility
Kim [92], 2016, South Korea	To examine the effects of the Buddhist ontology focused meditation programme on the psychological health and well-being of mothers of children with ASD	Mixed methods, repeated measures, within-subjects/questionnaires, interview/ANOVA, content analysis Affect: Positive and Negative Affect Schedule (PANAS; Watson, Clark, and Tellegen [93]) Depression: Depression, Anxiety, and Stress Scale (DASS; Lovibond, and Lovibond [94]) Anxiety: State-Trait Anxiety Inventory (STAI; Spielberger [57]) Cognitions and automatic thoughts: Metacognitions Questionnaire (MCQ; Cartwright-Hatton and Wells [95]) Depression: Beck Depression Inventory II (BDI-II; Beck, Steer, and Brown [52])	*n* = 9 mothers	A statistically significant improvement was found in psychological health well-being and positive affect. No changed was noted in anxiety or negative affect. Participants reported that the intervention helped them in relieving feelings of guilt that they had done wrong to accumulate bad karma and they were more aware of trying to accept people and things as they are.
Lovell et al. [96], 2016, UK	To examine the effectiveness of an intervention on reducing psychological distress in caregivers through written emotional disclosure regarding the benefits of caregiving	Experimental design/questionnaires, written essays/univariate ANOVA, chi square Depression: Hospital Anxiety and Depression Scale (HADS; Zigmond and Snaith [97]) Perceived stress: Perceived Stress Scale (PSS; Cohen and Williamson [73])	*n* = 33 (28 mothers, 5 fathers) treatment group: *n* = 16, control group: *n* = 17	Anxiety scores for parent carers in the benefit-finding condition were less likely to be in the clinical range 3-months post intervention
Nguyen et al. [98], 2016, United States	To determine if distress is reduced in mothers of children recently diagnosed with ASD following a problem-solving skills training program	Within-group, repeated measures/questionnaires/Mixed model analysis for repeated measures Depression: Beck Depression Inventory-Revised (BDI-R; Beck, Steer, and Brown [52]) Mood: Profile of Mood States (POMS; McNair et al. [99]) Psychological distress: Impact of Events Scale-Revised (IES-R; Creamer, Bell, and Failla [100])	*n* = 24 mothers	Mothers increased their effectiveness to solve problems and experienced fewer depressive symptoms over the course of the study, less post-traumatic stress symptoms and less disturbance of mood was reported at 3-month follow-up but problem-solving skills had decreased
Niimomi et al. [101], 2016, Japan	To determine the effectiveness of a parenting support group program in reducing parental stress and improving quality of life	Within-subjects design/questionnaires/Repeated-measure multivariate analysis Parenting stress: Parenting Stress Index-Short Form (PSI-SF; Abidin [69]) Quality of life: World Health Organization Quality of Life 26 item (WHOQOL-26; WHOQOL Group [102])	*n* = 24 mothers	A significant increase in quality of life was reported among participants at the conclusion of the program and at 3-months post follow-up. No change was noted in reducing parental stress.
Patra et al. [103], 2015, India	To develop a psycho-educational intervention for parents and to determine it’s impact on parent stress and knowledge of ASD	Mixed design, within-subjects/questionnaires, interview/Wilcoxon-signed rank test, nominal group technique Parenting stress: Family Interview for Stress and Coping (FISC-MR; Girimaji, et al. [104])	*n* = 36 married parents (18 mothers, 18 fathers)	Parents’ social stress and total stress decreased by the conclusion of intervention.
Ryan and Ahman [105], 2016, Jordan	To examine effectiveness of brief mindfulness-based intervention on parent carers perceived quality of life (QoL) and positive stress reappraisal	Quasi-experimental with non-equivalent control group/questionnaires/Paired sample *t*-test, pre-post test Coping: Cognitive Emotion Regulation Questionnaire (CERQ; Garnefski, Kraaij, and Spinhoven [106]) Mindfulness: Mindful Attention Awareness Scale (MAAS; Brown and Ryan [71]) Quality of life: World Health Organization Quality of Life-Brief Version (WHOQOL-BREF; WHOQOL Group [107])	*n* = 110 (73 mothers; 31 fathers) treatment group: *n* = 52 control group: *n* = 52	Physical health and environmental health domains of QoL were not significant between groups, but there was a significant difference on psychological health, social relationship health, and overall QoL among participants in the mindfulness intervention group
Ruiz-Robledillo et al. [108], 2015, Spain	To assess the effects of a mindfulness intervention on the mood disturbances and health complaints among parent caregivers in comparison to non-caregivers	Quasi-experiemental, within-group, between-group Anger; State-Trait Anger Expression Inventory-2 (STAXI-2; Miguel-Tobal et al. [109]) Anxiety: State-Trait Anxiety Inventory (STAI; Spielberger [57]) Caregiver stress or burden: Zarit Burden Inventory (ZBI; Zarit et al. [110]) Depression: Beck Depression Inventory (BDI; Beck and Steer [111]) General health: General Health Questionnaire-28 item (GHQ-28; Goldberg and Williams [53]) Mood: Profile of Mood States (POMS; McNair et al. [99]) Somatic symptoms: Somatic Symptoms Scale-Revised (Spanish Version) (ESS-R; Sandin and Chorot [112])	*n* = 13 (13 mothers; 1 father) Caregiver group: *n* = 6 (5 mothers, 1 father) Non-Caregiver group: *n* = 7 (7 mothers)	A significant reduction in mood disturbances and afternoon cortisol levels occurred for all participants, but were more pronounced in the caregiver group of parents. All participants reported a decrease in depressive and somatic symptoms at the end of the program and improved self-perceived general health.
Samadi et al. [113], 2012, Iran	To determine the effectiveness of a short group-based support course designed to increase parent’s knowledge of ASD and interventions to promote child’s development, boost parent’s confidence and sense of empowerment, and encourage parents to provide informal support to each other	Mixed design included pre-post, crossover design, within-group differences, between-group differences, questionnaires, interviews, paired *t*-tests, independent *t*-tests, content analysis Coping: Coping Styles Questionnaire (CSQ; Roger, Jervis and Najarian [114]) Family functioning: McMaster Family Assessment Device (FAD; Epstein, Baldwin and Bishop [85]) General health: General Health Questionnaire-28 item (GHQ-28; Goldberg and Williams [53]) Parenting stress: Parenting Stress Index-Short Form (PSI-SF; Abidin [69])	*n* = 37 (24 mothers, 13 fathers)	Intervention resulted in significant improvements on parental ratings of health, stress, and family functioning, and a significant increase in use of problem-focused coping strategies up to 4-months post intervention
McConkey and Samadi [115], 2013, IranNOTE: This study is a 12-month follow-up study to Samadi et al. [113] and thus was not counted as a separate intervention study in the overall number of studies.	To determine the extent of informal support among Iranian parents who had a child with ASD following a group-based training course and if indicators of parental well-being and use of problem-focused and emotional focused coping strategies were maintained 12 months post-intervention	Mixed design included pre-post, crossover design, within-group differences, between-group differences/questionnaires, interviews/paired *t*-tests, independent *t*-tests, thematic analysis Coping: Coping Styles Questionnaire (CSQ; Roger, Jervis and Najarian [114]) Family functioning: McMaster Family Assessment Device (FAD; Epstein, Baldwin and Bishop [85]) Family health: General Health Questionnaire-28 item (GHQ-28; Goldberg and Williams [53]) Parenting stress: Parenting Stress Index-Short Form (PSI-SF; Abidin [69])	*n* = 28 (17 mothers, 11 fathers)	Improved family functioning and better health of parents was maintained up to 12 months post-intervention, particularly among those parents who had maintained contact with others in the initial group-based intervention; child-related stress levels returned to baseline regardless if contact had been maintained; problem-focused coping strategies were not maintained 12 months-post intervention
Tongue et al. [116], 2006, Australia	To determine the impact of a parent education and behavior management intervention (PEBM), and a parent education and counseling intervention (PEC) on the mental health and adjustment of parent carers with preschool children with autism	Experimental design/questionnaires/Analysis of Covariance Family functioning: McMaster Family Assessment Device (FAD; Epstein, Baldwin and Bishop [85]) Family health: General Health Questionnaire-28 item (GHQ-28; Goldberg and Williams [53]) Parenting stress: Parenting Stress Thermometer [116] [visual analogue rating of general stress from 0 (none) to 4 (very, very much)]	*n* = 105 (info on gender of sample not reported) PEBM group: *n* = 35, PEC group: *n* = 35, Control group: *n* = 35	Both the PEBM and PEC interventions contained the same educational material, however the PEBM intervention included sessions that were skills based and action oriented while the PEC intervention emphasized nondirective, interactive discussion and counseling. There were no significant differences between the outcomes of the PEBM and PEC groups-both interventions reduced overall symptoms of parental distress, although the PEC intervention reduced the depressive symptoms in a larger percentage of parents.
Zhang et al. [117], 2014, China	To evaluate the effectiveness of a group-based solution-focused brief therapy on promoting post-traumatic growth	Experimental design/questionnaires/independent samples *t*-test Post-traumatic growth: Post-Traumatic Growth Inventory-Chinese version (PTGI-C; Tedeschi and Calhoun [118])	*n* = 45 mothers; Treatment groups = 20 (2 groups of 10), Control group = 25	Post-traumatic growth was significantly higher among mothers in the intervention groups compared to control group. The difference was maintained at a 3-months follow-up.

**Table 2:** Breakdown of studies into themes.

Theme and Subthemes	47	50	57	62	66	67	71	74	77	79	80	84	91	94	98	100	104	106	108	111	116/118	119	120
Importance of social support																							
Informal networks	+		+		+												+	+			+	+	
Reduced isolation	+		+	+	+																+	+	
Validation by peers	+		+	+	+					+				+							+		+
Skill development																							
Acceptance/Stress management	+	+		+			+		+				+	+	+				+	+			
Problem-solving	+	+		+	+	+	+					+				+							+
Gaining knowledge																							
ASD					+			+		+								+			+		
Resources, services				+				+			+							+					

+ indicates theme found or reported in article.
